# Blinatumomab versus historical standard therapy in pediatric patients with relapsed/refractory Ph-negative B-cell precursor acute lymphoblastic leukemia

**DOI:** 10.1038/s41375-020-0770-8

**Published:** 2020-02-24

**Authors:** Franco Locatelli, James A. Whitlock, Christina Peters, Christiane Chen-Santel, Victoria Chia, Robyn M. Dennis, Kenneth M. Heym, Aaron J. Katz, Michael A. Kelsh, Richard Sposto, Huakang Tu, Catherine A. Tuglus, Anupam Verma, Luciana Vinti, Jennifer J. Wilkes, Nathalya Zubarovskaja, Gerhard Zugmaier, Arend von Stackelberg, Weili Sun

**Affiliations:** 1 Department of Pediatric Hematology/Oncology and of Cell and Gene Therapy, IRCCS Bambino Gesù Children’s Hospital, Rome, Sapienza University of Rome, Rome, Italy; 2 The Hospital for Sick Children/University of Toronto, Toronto, ON Canada; 3 St. Anna Children’s Hospital, Vienna, Austria; 4 Department of Paediatric Oncology/Hematology/BMT, Charité Universitäts Medizin Berlin, Berlin, Germany; 5 Amgen Inc, Thousand Oaks, CA USA; 6 Nationwide Children’s Hospital, The Ohio State University, Columbus, OH USA; 7 Cook Children’s Medical Center, Fort Worth, TX USA; 8 Department of Epidemiology and Biostatistics, University of California, San Francisco, CA USA; 9 Children’s Hospital Los Angeles and Keck School of Medicine, University of Southern California, Los Angeles, CA USA; 10 Primary Children’s Hospital, Pediatric Hematology/Oncology, University of Utah, Salt Lake City, UT USA; 11 Center for Clinical and Translational Research, Seattle Children’s Hospital and Research Institute, Seattle, WA USA; 12 Department of Pediatrics, University of Washington, Seattle, WA USA; 13 Amgen Research, Munich, Germany; 14 City of Hope National Medical Center, Pediatric Hematology Oncology, Duarte, CA USA; 15 Present Address: Janssen Pharmaceutica, Beerse, Belgium

**Keywords:** Cancer, Acute lymphocytic leukaemia

## To the Editor:

Relapse and, less frequently, refractoriness to front-line therapy are the main causes of treatment failure in childhood B-cell precursor acute lymphoblastic leukemia (BCP-ALL), occurring in 15–20% of patients [1, 2]. Prognosis after relapse depends primarily on the time elapsing between diagnosis and relapse, site of relapse, and disease immunophenotypes [2]; unfortunately, many of these patients further relapse despite receiving allogeneic hematopoietic stem cell transplantation (allo-HSCT) [3].

Blinatumomab, a bispecific T-cell engager antibody construct, directs CD3-positive effector-memory T lymphocytes towards CD19-positive cells, triggering cell death of the latter [4]. Efficacy of blinatumomab in pediatric patients with relapsed/refractory (R/R) BCP-ALL has been demonstrated in an international phase 1/2, single-arm study (NCT01471782) [4].

R/R pediatric ALL is rare; consequently, most studies are single-arm and limited by small population sizes. Complete remission (CR) rates for pediatric patients in first or more advanced relapse vary from 8 to 75% [2, 5–9]. This variation can be attributed to differences among patient characteristics, sample sizes, and definition of CR used [2, 5–9].

For rare diseases, one approach to estimate treatment efficacy is to identify appropriate control populations with similar characteristics [9, 10]. Such a comparison has been undertaken for adult patients with Ph-negative R/R BCP-ALL from a single-arm study of blinatumomab with a historical dataset [11], but not for children.

We analyzed the blinatumomab phase 1/2 study [4] in comparison with three historical comparator groups from North America, Australia, and Europe. Propensity score (PS) analyses, along with a more conventional weighted analysis, evaluated two endpoints: overall survival (OS) and CR. The PS approach aims to create a balance between blinatumomab-treated subjects and historical comparator subjects with respect to multiple prognostic clinical factors.

Patients (<18 years) who had received intensive polychemotherapies with curative intent for R/R ALL in the time period 2005–2013 were included in this analysis by three historical comparator groups. The TACL study (group 1), conducted in 24 pediatric centers in the USA, Canada, and Australia, collected data on patients with R/R or relapsed after HSCT BCP-ALL (≤21 year-old) who received standard-of-care (SOC) chemotherapy 2005–2013. Only data from patients aged <18 years at time of earliest qualifying treatment failure were included in this analysis [9]. Two EU historical study groups provided data collected retrospectively from existing databases from Austria and Germany BFM (Berlin–Frankfurt–Münster) (group 2) and the Italian AIEOP (Associazione Italiana di Ematologia e Oncologia Pediatrica) (group 3) study groups.

Patient characteristics and endpoint definitions in the historical comparator studies were aligned to those used in the blinatumomab study [4]. Patients with Ph-negative R/R BCP-ALL with one of the following earliest qualifying events were selected: refractory to SOC induction/reinduction chemotherapy, relapse after allo-HSCT, or ≥2nd bone marrow (BM) relapse. The last qualifying treatment was used for these analyses, because they were more comparable with the blinatumomab study population. At the time of treatment for R/R disease, patients were required to have >25% leukemia BM blasts, without central nervous system involvement at time of qualifying event and to have had no previous, or current, treatment with blinatumomab. Information was documented from the date of initial ALL diagnosis through the date of R/R disease until the date of death or last follow-up.

Patients with different outcome measures in historical comparator groups are summarized in Supplementary Fig. 1. CR with or without full hematological recovery was defined in accordance with the blinatumomab study [4]. CR with full peripheral blood count recovery (CR-full) was defined as CR with ANC ≥ 0.5 × 10^9^/l and platelet count ≥100 × 10^9^/l. CR-full was not available for the BFM dataset. Follow-up time for OS was from the date of the start of the last salvage therapy, or date of last relapse if salvage date was not available, to date of death or last follow-up. Patients lost to follow-up were censored at the last known follow-up date.

Two statistical methods (i.e., conventional weighted analysis and PS-weighted comparative analysis) were applied to quantitatively evaluate the effect of blinatumomab on OS and CR rates, while adjusting for important risk factors for both endpoints. The main strata used were the nature of refractory disease/relapse (disease status), BM blasts, and time from prior treatment (Supplementary Appendix A). The 95% CIs were estimated by bootstrapping (Supplementary Appendix B). Weighting by PS analysis allowed estimation of treatment effect and CIs, while adjusting for differences in multiple data sources [12, 13]. The propensity to be treated was estimated via logistic regression model, using the patient’s treatment status as the outcome and a stepwise selection method to select among main effects and two-way interactions of the following covariates (see also the appendix): age; gender, region; previous allo-HSCT; number of previous salvage therapies; time since last therapy or allo-HSCT; percentage of BM blasts before starting salvage therapy; refractoriness to previous therapy; 11q23 abnormalities. The PS-weighted CR or OS analysis was performed using a Cox proportional hazard model or logistic regression model weighted with stabilized inverse probability of treatment weights (IPTW) derived from the predicted PS. The models included as independent variables patients’ treatment status and any covariates not sufficiently balanced by the PS weighting and estimated odd-ratios (OR) or hazard-ratios (HR) for treatment effects.

Baseline patient demographics and clinical characteristics among historical comparator groups and blinatumomab-treated population are shown in Table 1. In the blinatumomab-treated population, 70% of patients had relapsed <6 months from the last prior treatment compared with 46% in the combined historical groups.

**Table 1:** Baseline patient demographics and clinical characteristics in the historical comparator and blinatumomab studies.

	Combined historical dataset (*n* = 352)	TACL study group (*n* = 154)	BFM study group^a^ (*n* = 124)	AIEOP study group (*n* = 74)	Blinatumomab study group (MT103–205) (*n* = 70)
Mean age (SD), years	9.4 (4.7)	10.1 (4.8)	9.2 (4.5)	8.0 (4.4)	8.3 (5.0)
Age group, *n* (%)					
< 2 years	16 (5)	9 (6)	3 (2)	4 (5)	10 (14)
2–6 years	67 (19)	23 (15)	25 (20)	19 (26)	20 (29)
7 to < 18 years	269 (76)	122 (79)	96 (77)	51 (69)	40 (57)
Gender, *n* (%)					
Male	202 (57)	75 (49)	77 (62)	50 (68)	47 (67)
Female	150 (43)	79 (51)	47 (38)	24 (32)	23 (33)
Disease status, *n* (%)					
No HSCT, ≥2 relapses	84 (24)	51 (33)	20 (16)	13 (18)	8 (11)
No HSCT, refractory disease	75 (21)	45 (29)	24 (19)	6 (8)	22 (32)
Relapsed after HSCT	193 (55)	58 (38)	80 (65)	55 (74)	40 (57)
Previous HSCT, *n* (%)					
Yes	193 (55)	58 (38)	80 (65)	55 (74)	40 (57)
No	159 (45)	96 (62)	44 (35)	19 (26)	30 (43)
Number of prior lines of treatment, *n* (%)					
1	38 (11)	8 (5)	12 (10)	18 (25)	8 (11)
2	222 (64)	91 (59)	81 (66)	50 (70)	41 (59)
>2	88 (25)	55 (36)	30 (24)	3 (4)	21 (30)
BM blasts at the start of salvage treatment, *n* (%)					
<50%	50 (14)	30 (19)	12 (10)	8 (11)	18 (26)
≥50%	302 (86)	124 (81)	112 (90)	66 (89)	52 (74)
Time since previous treatment^b^, *n* (%)					
≤6 months	163 (46)	78 (51)	59 (48)	26 (35)	49 (70)
>6 months	189 (54)	76 (49)	65 (52)	48 (65)	21 (30)
Cytogenetics, *n* (%)					
Normal	152 (43)	48 (31)	51 (41)	53 (72)	22 (31)
*11q23* (*MLL* gene) rearranged	33 (9)	19 (12)	10 (8)	4 (5)	8 (11)
* Tel/AML-1*	13 (4)	3 (2)	6 (5)	4 (5)	6 (9)
Other abnormalities	80 (23)	63 (41)	13 (10)	5 (7)	28 (40)
Unknown	74 (21)	21 (14)	44 (35)	8 (11)	6 (9)

Data shown are *n* (%), unless otherwise stated.

*AIEOP* Associazione Italiana di Ematologia e Oncologia Pediatrica, *BFM* Berlin–Frankfurt–Münster, *BM* bone marrow, *HSCT* hematopoietic stem cell transplantation, *MLL* mixed lineage leukemia, *SD* standard deviation, *TACL* Therapeutic Advances in Childhood Leukemia and Lymphoma, *TEL/AML-1* t (12:21)(p13:q22) fusion transcript.

^a^The Austria database from BFM included patients who underwent allogeneic HSCT, whereas the Germany database from BFM included relapsed patients who were not transplanted.

^b^Chemotherapy or HSCT.

Unweighted proportions of CR-full (95% CI) in the combined TACL/AIEOP, TACL alone, and AIEOP alone groups are shown in Supplementary Table 1. CR-full (95%CI) values in the combined TACL/AIEOP group were 10% (5–14), 11% (6–15), and 9% (5–12) when weighted by disease status, BM blast percentage at treatment start, and time since previous treatment, respectively (Table 2) The corresponding CR proportions with/without peripheral blood count recovery (95% CI) in the combined TACL/BFM /AIEOP group were 44% (38–50), 48% (42–54), and 42% (36–47). The stratum-specific CR proportions with/without peripheral blood count recovery were higher in the BFM group than in the AIEOP and TACL groups for patients with refractory disease and those who had experienced ≥2 relapses (Supplementary Table 1).

**Table 2:** (a) Complete remission and median overall survival weighted to blinatumomab study data, and (b) propensity score weighted comparative analysis on complete remission and overall survival.

(a) Conventional weighted analysis								
	Combined historical comparator group		TACL study group		BFM study group		AIEOP study group	
*Weighted by disease status*								
CR with full peripheral blood count recovery (weighted CR proportion, % (95% CI))	10 (5–14)		9 (3–14)		NA		14 (1–24)	
CR with or without full peripheral blood count recovery (weighted CR proportion, % (95% CI))	44 (38–50)		43 (34–51)		52 (43–61)		37 (22–51)	
Combined weighted median OS (months (95% CI))	5.9 (5.0–6.7)		6.6 (2.6–8.4)		6.3 (4.0–8.0)		5.3 (1.5–7.2)	
*Weighted by bone marrow blasts at start of salvage treatment*								
CR with full peripheral blood count recovery (weighted CR proportion, % (95% CI))	11 (6–15)		9 (4–13)		NA		15 (4–25)	
CR with or without full peripheral blood count recovery (weighted CR proportion, % (95% CI))	48 (42–54)		46 (37–54)		57 (49–67)		43 (31–56)	
Combined weighted median OS (months (95% CI))	6.2 (4.3–7.1)		5.9 (3.3–7.1)		7.5 (0.0–10.9)		6.3 (4.9–10.1)	
*Weighted by time since previous treatment (chemotherapy or HSCT)*								
CR with full peripheral blood count recovery (weighted CR proportion, % (95% CI))	9 (5–12)		8 (3–12)		NA		12 (0–20)	
CR with or without full 2peripheral blood count recovery (weighted CR proportion, % (95% CI))	42 (36–47)		40 (32–48)		46 (36–56)		27 (14–38)	
Combined weighted median OS (months (95% CI))	5.5 (3.8–6.1)		6.3 (3.1–8.1)		6.0 (3.9–7.3)		4.7 (1.0–6.0)	

(b) Propensity score weighted comparative analysis								
	All study groups		TACL		BFM		AIEOP	
	Control (*n* = 352)	Blinatumomab (*n* = 70)	Control (*n* = 154)	Blinatumomab (*n* = 70)	Control (*n* = 124)	Blinatumomab (*n* = 70)	Control (*n* = 74)	Blinatumomab (*n* = 70)
CR with full peripheral blood count recovery								
Odds ratio (95% CI)	1.82 (0.74–4.51)^a^		2.44 (0.87–6.85)		N/A		4.94 (1.33–18.36)	
CR with or without full peripheral blood count recovery								
Odds ratio (95% CI)	0.67 (0.29–1.55)		0.63 (0.29–1.35)		0.50 (0.23–1.10)		1.87 (0.68–5.20)	
Overall survival								
Hazard ratio (95% CI)	0.65 (0.44–0.94)		0.86 (0.53–1.38)		0.82 (0.48–1.41)		0.50 (0.28–0.90)	

Only patients in the TACL and AIEOP datasets had peripheral blood count recovery. 86% (195/228) of the patients in TACL and AIEOP had peripheral blood counts. The stratum percentage weight for estimates is based on the Blincyto Study Group (MT103–205, *n* = 70).

For the CR with full peripheral blood count recovery group the combined comparator group includes TACL and AIEOP only. For the CR with or without full peripheral blood count recovery group the combined comparator group includes TACL, BFM, and AIEOP.

The propensity analysis utilized stabilized IPTW.

These data only include AIEOP and TACL.

*AIEOP* Associazione Italiana di Ematologia e Oncologia Pediatrica, *BFM* Berlin–Frankfurt–Münster, *CI* confidence interval, *CR* complete remission regardless of peripheral blood count recovery, *CR-full* complete remission with full recovery of peripheral blood counts, *HSCT* hematopoietic stem cell transplantation, *N* number of patients with data available to assess CR-full, *n* number of patients achieving CR-full, *NA* not available, *SD* standard deviation, *TACL* Therapeutic Advances in Childhood Leukemia and Lymphoma.

^a^Derived using the propensity score from the full data.

Median OS (95% CI) in the combined historical dataset was 6.2 months (5.1–7.2) (Supplementary Fig. 2A). Median OS was longer in the BFM group than in the AIEOP or TACL groups (Supplementary Fig. 2B). As published previously [4], the median OS (95% CI) in the blinatumomab study was 7.5 months (4.0–11.8) (Supplementary Fig. 3). Median OS estimates in the combined comparator group were 5.9, 6.2, and 5.5 months when weighted by disease status, BM blast percentage at treatment start, and time since previous treatment, respectively (Table 2). Median OS was longer for patients who had <50% blast cells than for those who had ≥50% blast cells at the start of salvage treatment (Supplementary Table 2). OS was shortest in patients with 11q23 abnormalities (3.3 months), and in those <6 year-old (Supplementary Fig. 4). For patients who had relapsed >6 months from last treatment, median OS was 9.3 months versus 3.9 months for those who had relapsed sooner (Supplementary Table 2).

In standardized IPTW, patients in the blinatumomab group were almost twice as likely to achieve a CR-full rate as the combined historical control group (OR, 1.82; 95% CI, 0.74–4.51). The HR for death with blinatumomab group versus historical controls was 0.65 (95% CI, 0.44–0.94) (Table 2).

Through historical comparator data from pediatric patients with R/R BCP-ALL and application of two analytical approaches, it was possible to compare the efficacy of blinatumomab from a single-arm, phase 1/2 study with that of historical SOC therapy. Single-agent blinatumomab treatment was associated with longer OS and a trend for higher CR-full in comparison with SOC chemotherapy, suggesting that the agent compares favorably with historical approaches.

We acknowledge that this study may have limitations: the weighted analysis relies on categorization by prognostic variables and stratifying by prognostic factors may not be sufficient for controlling confounding factors. Differences in data availability and collection among study populations can result in the exclusion of potentially important confounders in the PS model (e.g., physician’s reasons for treating patients with blinatumomab versus chemotherapy). Conclusions of propensity-adjusted analyses are limited by availability of overlapping covariates in the three study datasets. Finally, the limited sample size could reduce the power to detect clinically meaningful differences between groups. Nonetheless, this study has several strengths. Data were included from patients across six countries worldwide; pooling these data removed some of the noise observed when datasets were considered individually. Stratified and weighted analyses were used at the patient level to provide optimal data summaries.

This study revealed differences in outcomes by important stratifying factors: in the combined subgroups analyses, median OS was shortest in patients <6 years, in those with 11q23 abnormalities, in those with refractory disease and who had received their last treatment line <6 months from the event qualifying for study-entry. Similar trends were observed in the blinatumomab cohort, except that younger patients appeared to respond better than older patients [4]. Defining age groups according to International Council of Harmonization guidelines [10], resulted into no difference in efficacy across age groups.

Altogether, these data provide support to the efficacy of blinatumomab in R/R BCP-ALL.

## Supplementary information

Supplementary Figures and Table legends

Supplementary Table 1

Supplementary Table 2

Supplementary Fig 1

Supplementary Fig 2

Supplementary Fig 3

Supplementary Fig 4

Appendix
