# Supplementary material for: Blinatumomab versus historical standard therapy in pediatric patients with relapsed/refractory Ph-negative B-cell precursor acute lymphoblastic leukemia
Source: Leukemia. 2020 Feb 24;34(9):2473–8. doi: 10.1038/s41375-020-0770-8 (PMC7449874; doi:10.1038/s41375-020-0770-8)
Supplement: Supplementary file 1 — Supplementary Figures and Table legends [file 41375_2020_770_MOESM1_ESM.docx]

**Fig. legends**

**Supplementary Fig. 1** Historical database analysis cohort. *AIEOP* Associazione Italiana di Ematologia e Oncologia Pediatrica, *BFM* Berlin–Frankfurt–Münster, *CR complete remission, OS overall survival, TACL Therapeutic Advances in Childhood Leukemia and Lymphoma.*

**Supplementary Fig. 2** Kaplan–Meier curves of OS in (A) the combined TACL/EU historical study group and (B) individual historical study groups. The date of death was missing for five patients; therefore the time to death was not evaluable in these patients. *AIEOP* Associazione Italiana di Ematologia e Oncologia Pediatrica, *BFM* Berlin–Frankfurt–Münster, *CI* confidence interval, *EU* European Union, *OS* overall survival, *TACL* Therapeutic Advances in Childhood Leukemia and Lymphoma.

**Supplementary Fig. 3** Kaplan–Meier curves of OS in the blinatumomab study.

**Supplementary Fig. 4** Kaplan–Meier curves of overall survival in the combined TACL/EU historical study group, by subgroup. The date of death was missing for five patients; therefore the time to death was not evaluable in these patients. *AIEOP* Associazione Italiana di Ematologia e Oncologia Pediatrica, *BFM* Berlin–Frankfurt–Münster, *CI* confidence interval, *EU* European Union, *OS* overall survival, *TACL* Therapeutic Advances in Childhood Leukemia and Lymphoma.

**Supplementary Table 1** Complete remission with full peripheral blood count recovery and regardless of peripheral blood count recovery by strata and weighted to blinatumomab study data

*AIEOP* Associazione Italiana di Ematologia e Oncologia Pediatrica, *BFM* Berlin–Frankfurt–Münster, *CI* confidence interval, *CR* complete remission regardless of peripheral blood count recovery, *CR-full* complete remission with full recovery of peripheral blood counts, *HSCT* hematopoietic stem cell transplantation, *N* number of patients with data available to assess CR-full, *n* number of patients achieving CR-full, *NA* not available, SD standard deviation, *TACL* Therapeutic Advances in Childhood Leukemia and Lymphoma.

Only patients in the TACL and AIEOP datasets had peripheral blood count recovery. 86% (195/228) of the patients in TACL and AIEOP had peripheral blood counts. The stratum percentage weight for estimates is based on the Blincyto Study Group (MT103-205, *n* = 70).

**Supplementary Table 2** Median overall survival and survival rate by strata, weighted by blinatumomab clinical data

*AIEOP* l’Associazione Italiana di Ematologia e Oncologia Pediatrica, *BFM* Berlin–Frankfurt–Münster, *CI* confidence interval, *CR* complete remission, *EU* European Union, *HSCT* hematopoietic stem cell transplantation, *NE* not estimable, *OS* overall survival, *TACL* Therapeutic Advances in Childhood Leukemia and Lymphoma. The stratum percentage weight for estimates is based on the Blincyto Study Group (MT103-205, *n* = 70)
