# Supplementary material for: Blinatumomab versus historical standard therapy in pediatric patients with relapsed/refractory Ph-negative B-cell precursor acute lymphoblastic leukemia
Source: Leukemia. 2020 Feb 24;34(9):2473–8. doi: 10.1038/s41375-020-0770-8 (PMC7449874; doi:10.1038/s41375-020-0770-8)
Supplement: Supplementary file 2 — Supplementary Table 1 [file 41375_2020_770_MOESM2_ESM.docx]

**Supplementary Table 1** Complete remission with full peripheral blood count recovery and regardless of peripheral blood count recovery by strata and weighted to blinatumomab study data

|  | Stratum (%) for weighted estimate | Combined historical comparator group | | TACL study group | | BFM study group | | AIEOP study group | |
| --- | --- | --- | --- | --- | --- | --- | --- | --- | --- |
|  |  | *n*/*N* | CR proportion, % (95% CI) | *n*/*N* | CR proportion, % (95% CI) |  | NA | *n*/*N* | CR proportion, % (95% CI) |
| *CR with full peripheral blood count recovery (combined comparator group includes TACL and AIEOP only)* | | | | | | | | | |
| Overall |  | 20/195 | 10 (6, 15) | 12/136 | 9 (5, 15) |  | NA | 8/59 | 14 (6, 25) |
| Disease status  Second or later relapse  Refractory disease  Relapse after HSCT  Weighted | 11.4  31.4  57.2 | 7/59  3/46  10/90 | 12 (5–23)  7 (1–18)  11 (6–20)  10 (5–14) | 5/47  2/40  5/49 | 11 (4–23)  5 (1–17)  10 (3–22)  9 (3–14) |  | NA | 2/12  1/6  5/41 | 17 (2–48)  17 (4–64)  12 (4–26)  14 (1–24) |
| Bone marrow blasts at start of salvage treatment  < 50%  ≥ 50%  Weighted | 25.7  74.3 | 4/33  16/162 | 12 (3–28)  10 (6–16)  11 (6–15) | 2/25  10/111 | 8 (1–26)  9 (4–16)  9 (4–13) |  | NA | 2/8  6/51 | 25 (3–65)  12 (4–24)  15 (4–25) |
| Time since previous treatment (chemotherapy or HSCT)  ≤ 6 months  6 months  Weighted | 70.0  30.0 | 6/90  14/105 | 7 (3–14)  13 (8–21)  9 (5–12) | 4/70  8/66 | 6 (2–14)  12 (5–23)  8 (3–12) |  | NA | 2/20  6/39 | 10 (1–32)  15 (6–31)  12 (0–20) |
| *CR with or without full peripheral blood count recovery (combined comparator group includes TACL, BFM, and AIEOP)* | | | | | | | | | |
|  | | | | | | | | | |
| Overall |  | 145/314 | 46 (41–52) | 65/143 | 45 (37–54) | 57/109 | 52 (43, 62) | 23/62 | 37 (25, 50) |
| Disease status  Second or later relapse  Refractory disease  Relapse after HSCT  Weighted | 11.4  31.4  57.2 | 47/80  30/70  68/164 | 59 (47–70)  43 (31–55)  42 (34–49)  44 (38–50) | 27/48  15/43  23/52 | 56 (41–71)  35 (21–51)  44 (31–59)  43 (34–51) | 17/20  13/21  27/68 | 85 (62–97)  62 (38–82)  40 (28–61)  52 (43–61) | 3/12  2/6  18/44 | 25 (6–57)  33 (4–78)  41 (26–57)  37 (22–51) |
| Bone marrow blasts at start of salvage treatment  < 50%  ≥ 50%  Weighted | 25.7  74.3 | 29/48  116/266 | 60 (45–74)  44 (38–50)  48 (42–54) | 13/28  52/115 | 46 (28–66)  45 (36–55)  46 (37–54) | 10/12  47/97 | 83 (52–98)  49 (38–59)  57 (49–67) | 6/8  17/54 | 75 (35–97)  32 (20–46)  43 (31–56) |
| Time since previous treatment (chemotherapy or HSCT)  ≤ 6 months  > 6 months  Weighted | 70.0  30.0 | 53/158  102/169 | 34 (26–42)  60 (53–68)  42 (36–47) | 22/74  43/69 | 30 (20–42)  62 (50–74)  40 (32–48) | 20/54  37/55 | 37 (24–51)  65 (53–79)  46 (36–56) | 4/22  20/42 | 18 (5–40)  48 (32–64)  27 (14–38) |

*AIEOP* Associazione Italiana di Ematologia e Oncologia Pediatrica, *BFM* Berlin–Frankfurt–Münster, *CI* confidence interval, *CR* complete remission regardless of peripheral blood count recovery, *CR-full* complete remission with full recovery of peripheral blood counts, *HSCT* hematopoietic stem cell transplantation, *N* number of patients with data available to assess CR-full, *n* number of patients achieving CR-full, *NA* not available, SD standard deviation, *TACL* Therapeutic Advances in Childhood Leukemia and Lymphoma.

Only patients in the TACL and AIEOP datasets had peripheral blood count recovery. 86% (195/228) of the patients in TACL and AIEOP had peripheral blood counts. The stratum percentage weight for estimates is based on the Blincyto Study Group (MT103-205, *n* = 70).
