# Supplementary material for: Blinatumomab versus historical standard therapy in pediatric patients with relapsed/refractory Ph-negative B-cell precursor acute lymphoblastic leukemia
Source: Leukemia. 2020 Feb 24;34(9):2473–8. doi: 10.1038/s41375-020-0770-8 (PMC7449874; doi:10.1038/s41375-020-0770-8)
Supplement: Supplementary file 3 — Supplementary Table 2 [file 41375_2020_770_MOESM3_ESM.docx]

**Supplementary Table 2** Median overall survival and survival rate by strata, weighted by blinatumomab clinical data

|  | | Stratum (%) for weighted estimate | | Combined TACL/BFM/AIEOP study group  (*n* = 352) | | TACL study group  (*n* = 154) | | BFM study group  (*n* = 124) | | AIEOP study group  (*n* = 74) | | | | |  |
| --- | --- | --- | --- | --- | --- | --- | --- | --- | --- | --- | --- | --- | --- | --- | --- |
| Median OS | |  | | *n* | Months (95% CI) | *n* | Months (95% CI) | *n* | Months (95% CI) | *n* | | Months (95% CI) | | | |
| Disease status | |  |  |  |  |  |  |  |  |  |  |  |  |  |  |
| Second or later relapse | | 11.4 | | 84 | | 9.0 (6.6–10.3) | 51 | 9.0 (5.9–17.2) | 20 | 10.3 (7.0–NE) | 13 | 4.5 (0.9–9.3) | |  |  |
| Refractory disease | | 31.4 | | 74 | | 3.9 (3.1–4.7) | 44 | 3.2 (2.4–4.7) | 24 | 5.1 (3.8–11.3) | 6 | 3.0 (0.5–NE) | |  |  |
| Relapsed after HSCT | | 57.2 | | 191 | | 6.4 (5.2–7.5) | 58 | 8.0 (4.9–14.3) | 80 | 6.1 (4.3–7.2) | 53 | 6.7 (3.9–12.2) | |  |  |
| Combined weighted median OS | | | |  | 5.9 (5.0–6.7) |  | 6.6 (2.6–8.4) |  | 6.3 (4.0–8.0) |  | | 5.3 (1.5–7.2) | | |  |
| Bone marrow blasts at start of salvage treatment | | | | | | | | | | | | | | |  |
| < 50% | | 25.7 | | 50 | 7.2 (4.5–12.2) | 30 | 4.5 (2.9–9.0) | 12 | 11.5 (6.8–NE) | 8 | | 9.6 (2.4–NE) | | |  |
| ≥ 50% | | 74.3 | | 299 | 5.9 (5.0–7.0) | 123 | 6.4 (5.0–8.6) | 112 | 6.1 (4.4–7.7) | 64 | | 5.1 (3.2–6.9) | | |  |
| Combined weighted median OS | | | |  | 6.2 (4.3–7.1) |  | 5.9 (3.3–7.1) |  | 7.5 (0.0–10.9) |  | | 6.3 (4.9–10.1) | | |  |
| Time since previous treatment (chemotherapy or HSCT) | | | | | | | | | | | | | |  |  |
| ≤ 6 months | | 70.0 | | 161 | 3.9 (3.1–4.5) | 77 | 3.2 (2.4–4.7) | 59 | 4.2 (3.0–5.2) | 25 | | 3.9 (2.9–6.4) | | |  |
| > 6 months | | 30.0 | | 188 | 9.3 (8.1–14.3) | 76 | 13.6 (8.4–24.4) | 65 | 10.3 (7.7–18.1) | 47 | | 6.8 (2.9–15.8) | | |  |
| Combined weighted median OS | | | |  | 5.5 (3.8–6.1) |  | 6.3 (3.1–8.1) |  | 6.0 (3.9–7.3) |  | | 4.7 (1.0–6.0) | | |  |
| 12-month OS proportion | |  | | *n* | Survival proportion (95% CI) | *n* | Survival proportion (95% CI) | *n* | Survival proportion (95% CI) | *n* | | Survival proportion (95% CI) | | |  |
| Disease status | |  |  |  |  |  |  |  |  |  |  |  |  |  |  |
| Second or later relapse | | 11.4 | | 84 | 38 (27–48) | 51 | 41 (27–54) | 20 | 38 (16–60) | 13 | | 23 (6–47) | | |  |
| Refractory disease | | 31.4 | | 74 | 19 (11–29) | 44 | 14 (5–26) | 24 | 29 (13–48) | 6 | | 17 (1–52) | | |  |
| Relapsed after HSCT | | 57.2 | | 191 | 35 (28–41) | 58 | 40 (27–53) | 80 | 28 (19–38) | 53 | | 39 (25–52) | | |  |
| Combined weighted OS proportion | | | |  | 30 (25–35) |  | 32 (23–40) |  | 30 (21–38) |  | | 30 (17–41) | | |  |
| Bone marrow blasts at start of salvage treatment | | | | | | | | | | | | | | |  |
| < 50% | | 25.7 | | 50 | 38 (25–52) | 30 | 31 (15–48) | 12 | 48 (18–72) | 8 | | 50 (15–77) | | |  |
| ≥ 50% | | 74.3 | | 299 | 31 (26–36) | 123 | 34 (25–42) | 112 | 28 (20–37) | 64 | | 32 (21–44) | | |  |
| Combined weighted OS proportion | | | |  | 33 (28–38) |  | 33 (25–41) |  | 33 (23–43) |  | | 36 (24–49) | | |  |
| Time since previous treatment (chemotherapy or HSCT) | | | | | | | | | | | | | | |  |
| ≤ 6 months | | 70.0 | | 161 | 16 (10–22) | 77 | 15 (8–24) | 59 | 15 (7–26) | 25 | | 19 (6–38) | | |  |
| > 6 months | | 30.0 | | 188 | 46 (39–53) | 76 | 51 (39–62) | 65 | 43 (31–55) | 47 | | 41 (27–55) | | |  |
| Combined weighted OS proportion | | | | | 25 (20–29) |  | 26 (19–32) |  | 24 (16–32) |  | | 26 (13–38) | | |  |
| 24-month OS proportion | |  | | *n* | Survival rate (95% CI) | *n* | Survival rate (95% CI) | *n* | Survival rate (95% CI) | *n* | | Survival rate (95% CI) | | |  |
| Disease status | |  |  |  |  |  |  |  |  |  |  |  |  |  |  |
| Second or later relapse | | 11.4 | | 84 | 35 (25–45) | 51 | 36 (23–50) | 20 | 38 (16–60) | 13 | | 23 (6–47) | | |  |
| Refractory disease | | 31.4 | | 74 | 16 (9–26) | 44 | 11 (4–23) | 24 | 25 (10–43) | 6 | | 17 (1–52) | | |  |
| Relapsed after HSCT | | 57.2 | | 191 | 23 (17–29) | 58 | 28 (17–40) | 80 | 21 (12–30) | 53 | | 20 (10–33) | | |  |
| Combined weighted OS proportion | | | | | 22 (17–27) |  | 24 (16–31) |  | 24 (15–32) |  | | 19 (7–29) | | |  |
| Bone marrow blasts at start of salvage treatment | | | | | | | | | | | | | | |  |
| < 50% | | 25.7 | | 50 | 34 (21–47) | 30 | 31 (15–48) | 12 | 38 (12–64) | 8 | | 38 (9–67) | | |  |
| ≥ 50% | | 74.3 | | 299 | 23 (18–28) | 123 | 25 (17–33) | 112 | 23 (15–31) | 64 | | 19 (10–30) | | |  |
| Combined weighted OS proportion | | | |  | 26 (21–31) |  | 26 (19–34) |  | 27 (17–36) |  | | 24 (12–36) | | |  |
| Time since previous treatment (chemotherapy or HSCT) | | | | | | | |  |  |  | |  | | |  |
| ≤ 6 months | | 70.0 | | 161 | 12 (7–18) | 77 | 13 (7–22) | 59 | 13 (6–24) | 25 | | 5 (0–20) | | |  |
| > 6 months | | 30.0 | | 188 | 35 (28–42) | 76 | 39 (28–50) | 65 | 34 (23–46) | 47 | | 29 (16–43) | | |  |
| Combined weighted OS proportion | | | |  | 19 (14–23) |  | 21 (15–27) |  | 20 (12–27) |  | | 12 (3–18) | | |  |

*AIEOP* l’Associazione Italiana di Ematologia e Oncologia Pediatrica, *BFM* Berlin–Frankfurt–Münster, *CI* confidence interval, *CR* complete remission, *EU* European Union, *HSCT* hematopoietic stem cell transplantation, *NE* not estimable, *OS* overall survival, *TACL* Therapeutic Advances in Childhood Leukemia and Lymphoma. The stratum percentage weight for estimates is based on the Blincyto Study Group (MT103-205, *n* = 70).
