# Supplementary figures and images for: Blinatumomab versus historical standard therapy in pediatric patients with relapsed/refractory Ph-negative B-cell precursor acute lymphoblastic leukemia
Source: Leukemia. 2020 Feb 24;34(9):2473–8. doi: 10.1038/s41375-020-0770-8 (PMC7449874; doi:10.1038/s41375-020-0770-8)

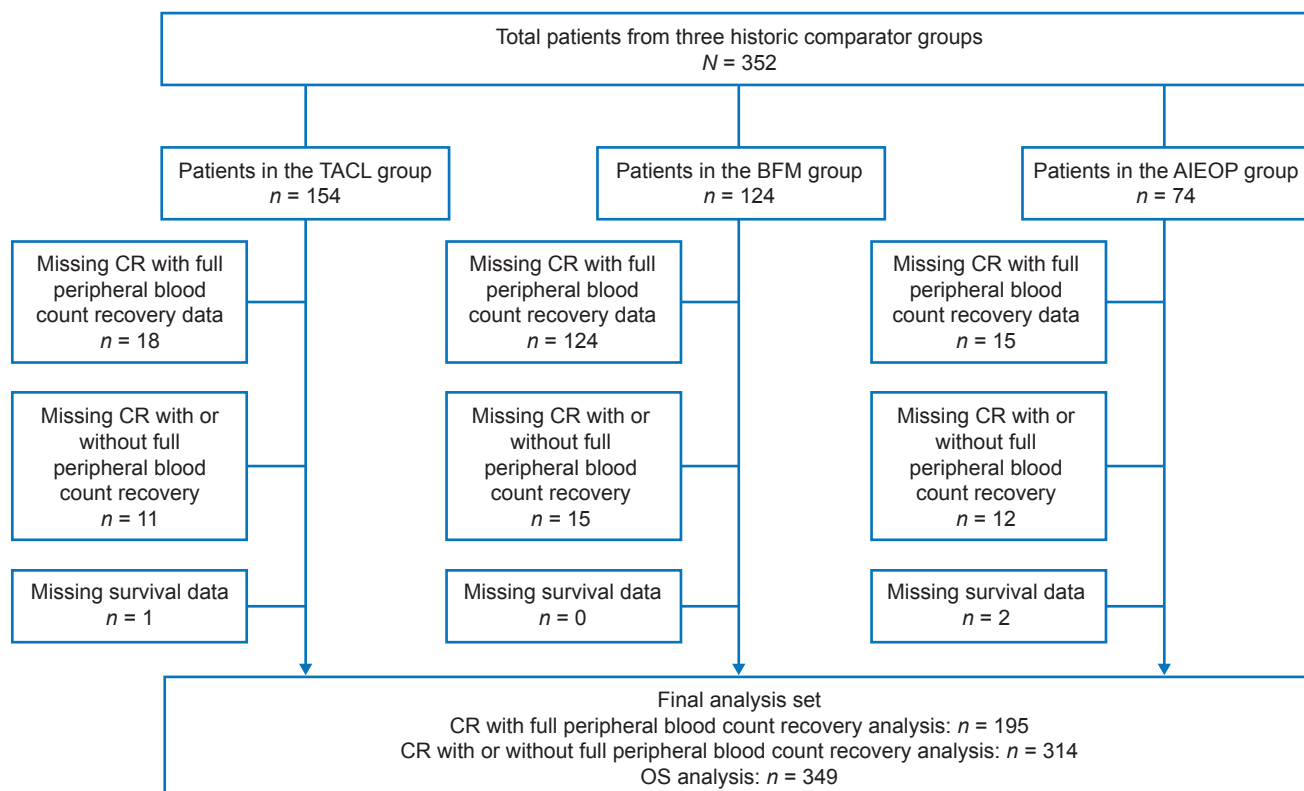

Supplement: Supplementary file 4 — Supplementary Fig 1 [file 41375_2020_770_MOESM4_ESM.pdf]

A

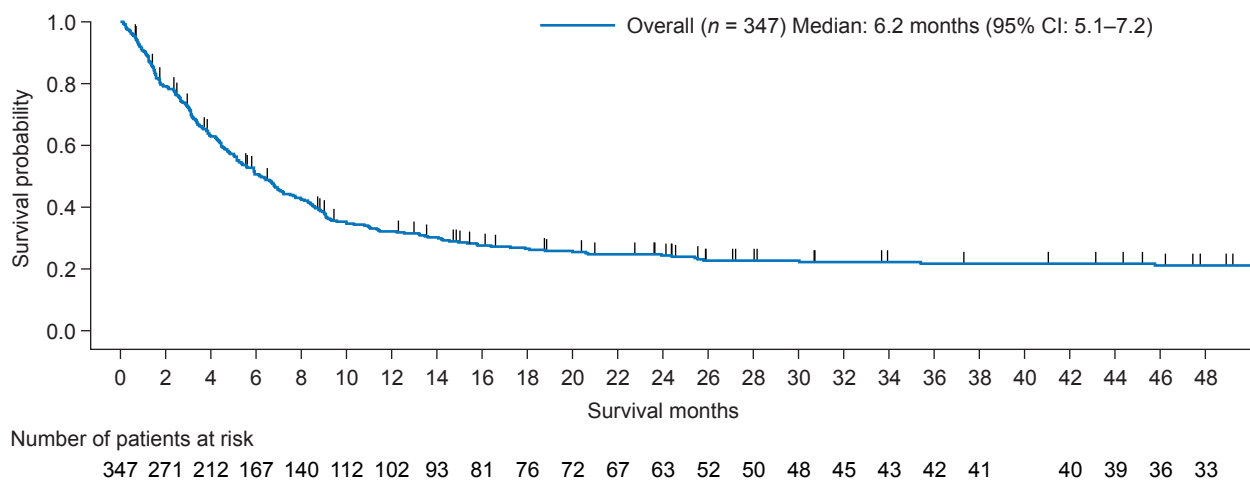

B

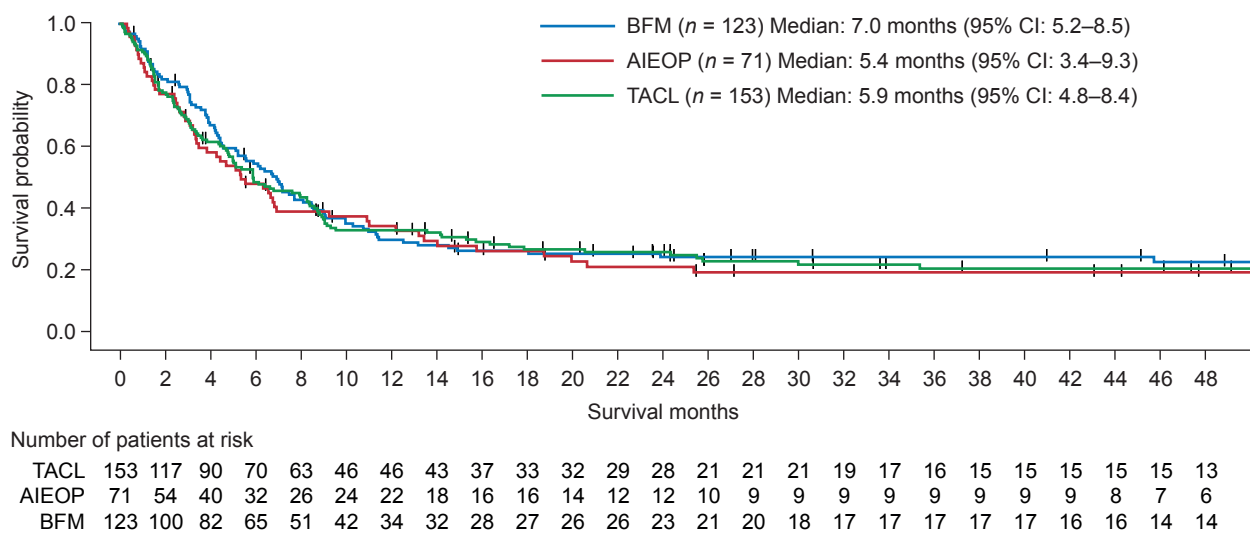

Supplement: Supplementary file 5 — Supplementary Fig 2 [file 41375_2020_770_MOESM5_ESM.pdf]

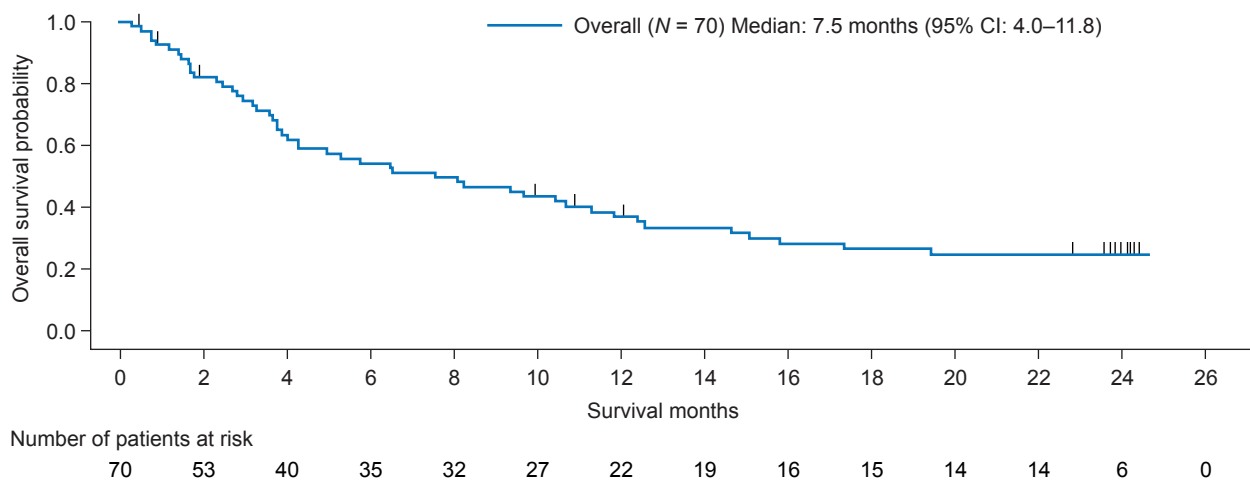

Supplement: Supplementary file 6 — Supplementary Fig 3 [file 41375_2020_770_MOESM6_ESM.pdf]
