# Supplementary material for: Blinatumomab versus historical standard therapy in pediatric patients with relapsed/refractory Ph-negative B-cell precursor acute lymphoblastic leukemia
Source: Leukemia. 2020 Feb 24;34(9):2473–8. doi: 10.1038/s41375-020-0770-8 (PMC7449874; doi:10.1038/s41375-020-0770-8)
Supplement: Supplementary file 7 — Supplementary Fig 4 [file 41375_2020_770_MOESM7_ESM.pdf]

A

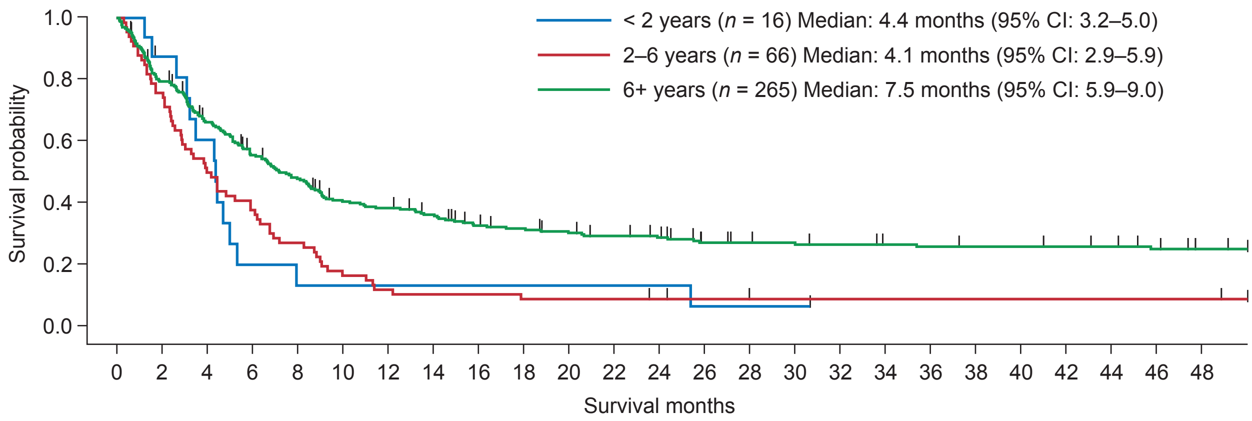

Number of patients at risk

	0	2	4	6	8	10	12	14	16	18	20	22	24	26	28	30	32	34	36	38	40	42	44	46	48
< 2 years	265	208	169	139	120	98	92	84	72	68	64	59	56	47	45	44	42	40	39	38	38	37	36	33	30
2–6 years	66	50	34	25	18	12	8	7	7	6	6	6	5	4	4	3	3	3	3	3	3	3	3	3	3
6+ years	16	13	9	3	2	2	2	2	2	2	2	2	2	1	1	1	0								

B

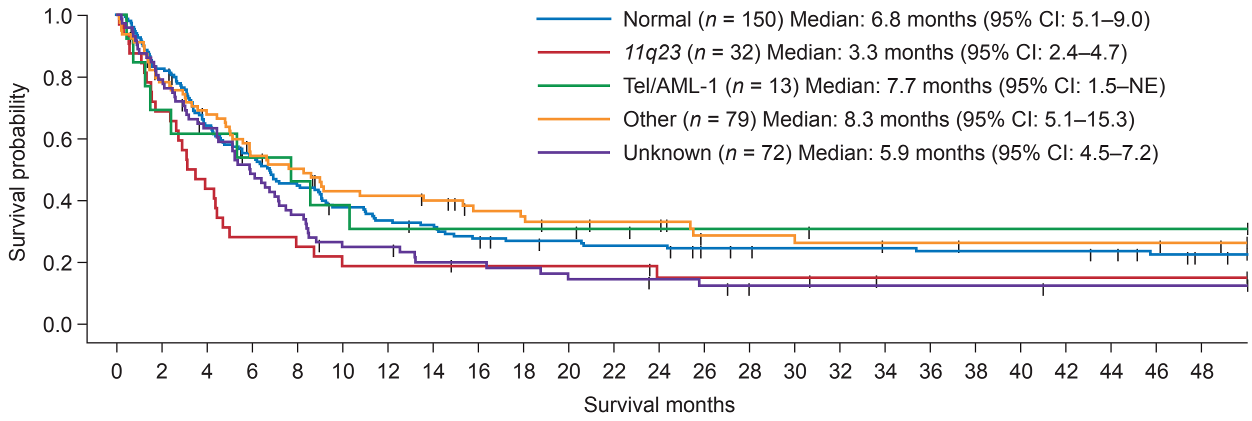

Number of patients at risk

	0	2	4	6	8	10	12	14	16	18	20	22	24	26	28	30	32	34	36	38	40	42	44	46	48
Normal	72	56	43	33	24	17	16	12	11	10	9	8	7	6	5	4	4	4	4	4	4	3	3	3	3
11q23	79	60	52	40	36	29	28	26	21	20	18	17	17	12	12	12	11	10	10	9	9	9	9	9	8
Tel/AML-1	13	9	8	7	6	5	4	4	4	4	4	3	2	2	2	2	1	1	1	1	1	1	1	1	1
Other	32	22	14	9	8	7	6	6	6	6	6	6	4	4	4	4	3	2	2	2	2	2	2	2	2
Unknown	150	123	94	77	65	53	47	44	38	35	34	32	32	28	27	26	26	26	25	25	25	25	24	21	19
