# Supplementary material for: Blinatumomab versus historical standard therapy in pediatric patients with relapsed/refractory Ph-negative B-cell precursor acute lymphoblastic leukemia
Source: Leukemia. 2020 Feb 24;34(9):2473–8. doi: 10.1038/s41375-020-0770-8 (PMC7449874; doi:10.1038/s41375-020-0770-8)
Supplement: Supplementary file 8 — Appendix [file 41375_2020_770_MOESM8_ESM.docx]

**Supplementary Appendix**

Appendix A Prognostic Factors Included in Weighting and Propensity Score Model

| **Prognostic Factor** | **Note** | **Reference** |
| --- | --- | --- |
| **Age at diagnosis** | Continuous | Vrooman *et al* 2016 |
|  |  |  |
| **Previous HSCT** | Yes | Fagioli *et al* 2013 |
|  | No |  |
| **Number of previous lines of salvage therapy** | 0, 1, 2, and > 2 | Sun *et al* 2018, Ko *et al* 2010, von Stackelberg *et al* 2011 |
| **Time since last therapy or HSCT (months)** | Continuous | Sun *et al* 2018 |
|  |  |  |
| **Percentage of bone marrow blasts before start of qualifying salvage therapy** | < 50% or ≥ 50% | Sun *et al* 2018,  Topp *et al* 2014 |
|  |  |  |
| **Refractoriness to previous therapy** | Yes | Sun *et al* 2018, Wei *et al* 2015, Locatelli *et al* 2012 |
|  | No |  |
| **11q23 abnormalities (mixed lineage leukemia [MLL] gene rearrangement** | Yes | Pieters *et al* 2007, Dreyer *et al* 2015 |
|  | No |  |
| **Sex** | Male, Female |  |
| **Geographical Region** | EU; |  |
|  | US/Canada/Australia; |  |
|  |  |  |
| Dreyer ZE, et al. Intensified chemotherapy without SCT in infant ALL: results from COG P9407 (Cohort 3). Pediatr Blood Cancer 2015;62:419–26; Fagioli F, et al. Hematopoietic stem cell transplantation for children with high-risk acute lymphoblastic leukemia in first complete remission: a report from the AIEOP registry. Haematologica 2013;98(8):1273-81; Ko RH, et al. Outcome of patients treated for relapsed or refractory acute lymphoblastic leukemia: A Therapeutic Advances in Childhood Leukemia Consortium study. J Clin Oncol 2010.;28:648-654; Locatelli F, et al. How I treat relapsed childhood acute lymphoblastic leukemia. Blood 2012;120:2807-2816; Pieters R, et al. A treatment protocol for infants younger than 1 year with acute lymphoblastic leukaemia (Interfant-99): an observational study and a multicentre randomised trial. Lancet. 2007; 370(9583):240-250; Sun W, et al. Outcome of children with multiply relapsed B-cell acute lymphoblastic leukemia: a therapeutic advances in childhood leukemia & lymphoma study. Leukemia 2018;32:2316–2325; Topp MS, et al. Phase II trial of the anti-CD19 bispecific T cell-engager blinatumomab shows hematologic and molecular remissions in patients with relapsed or refractory B-precursor acute lymphoblastic leukemia J Clin Oncol 2014;32:4134-4140; Vrooman LM et al. Treatment of Childhood Acute Lymphoblastic Leukemia: Prognostic Factors and Clinical Advances Current Hematologic Malignancy Reports. 2016; 11:385–394; von Stackelberg A, et al. Outcome of children and adolescents with relapsed acute lymphoblastic leukaemia and non-response to salvage protocol therapy: A retrospective analysis of the ALL-REZ BFM Study Group. 2011;47(1):90-7; Wei W, et al. Prediction of outcomes by early treatment responses in childhood T-cell acute lymphoblastic leukemia: a retrospective study in China. BMC Pediatr. 2015; 15:80 | | |

Appendix B

1. Bootstrapping method for 95% CI estimation

The following method was used to estimate the 95% CI for the combined stratum adjusted estimates of the endpoint:

1. Calculate the combined stratum adjusted estimate, *μ*_all_, of the summary statistic of interest which is the sum of the strata estimates weighted by the proportion of subjects observed in each stratum from the blinatumomab study.
2. Divide the analysis set into separate analysis sets based on stratum.
3. For each of the analysis sets, generate 200 samples with replacement with each sample being the size of the analysis set for that stratum.
4. Set together the bootstrap sample data sets and sort by replicate number (1–2000).
5. For each replicate, calculate the combined stratum adjusted estimate, *μ_i_*, *i* = 1–2000.
6. Calculate the absolute deviation between *μ_i_* and *μ*_all_, which is noted as *d_i_.*
7. Order *d_i_* from smallest to largest, which *d*_[1]_ ≤ *d*_[2]_ ≤ *d*_[3]_ ≤ ... ≤ *d*_[2000]_*.*
8. The 95% CI for the combined stratum adjusted estimate is (*μ*_all_ – *d*_[1950]_, *μ*_all_ – *d*_[50]_)*.*

The SAS code for creating the bootstrapping sample:

**data** bootsamples_stratum1;

do replicate = **1** to 2000;

do i = **1** to numrecs;

p = int(**1** + numrecs*(ranuni(**28374**)));

set stratum_1 point=p nobs=numrecs;

output;

end;

end;

stop;

run;

Note: *numrecs* is the background variable for the number of the records available within a stratum dataset in the *set* statement.
